# Children’s Cognitive and Emotional Processes in Adult Versus Child-Related Inter-Parental Conflicts

**DOI:** 10.5964/ejop.v15i4.1613

**Published:** 2019-12-19

**Authors:** Elena Camisasca, Sarah Miragoli, Paola Di Blasio

**Affiliations:** a Università Telematica e-Campus, Novedrate, Italy; b C.R.I.d.e.e., Department of Psychology, Catholic University of Milan, Milan, Italy; University of Wollongong, Wollongong, Australia

**Keywords:** inter-parental conflict, cognitive appraisals, distress, triangulation, internalizing and externalizing behaviors, children

## Abstract

In the literature, little attention has been paid to the specific impact of child-related versus adult-related inter-parental conflicts on children’s intrapersonal processes and adjustment. Aimed to advance knowledge on this topic, the cross-sectional study explores: 1) the predictive effects of the two forms of inter-parental conflicts on: a) children’s internalizing/externalizing behaviors and b) children’s cognitive appraisals, emotional distress, and triangulation; 2) the mediating role of children’s cognitive appraisals, emotional distress, and triangulation, in the association between adult-related vs child-related conflict and children’s adjustment. Seventy-five school-aged children and their parents completed measures of inter-parental conflict, cognitive, emotional and behavioral processes and child adjustment. The results indicated that: 1) higher levels of adult-related inter-parental conflict promoted children’s internalizing behaviors, through the mediation of perceived threat; 2) higher levels of child-related inter-parental discord promoted both children’s internalizing/externalzing behaviors, through the mediation of perceived threat and self-blame.

Although conflict is inevitable in every marriage, the frequency, intensity and content of discord vary across couples. When parents manage their divergences in positive ways, by displaying verbal and physical affection, problem solving and support, the conflict is said to be constructive (Goeke-Morey, Cummings, Harold, & Shelton, 2003; McCoy, Cummings, & Davies, 2009; McCoy, George, Cummings, & Davies, 2013). In contrast, destructive conflict is characterized by chronic, unresolved and intense levels of hostility. This type of discord undermines children’s sense of security (Camisasca, Miragoli, Di Blasio, & Grych, 2017; Cummings, Goeke-Morey, & Papp, 2003; Goeke-Morey et al., 2003), making them more vulnerable to development of adjustment problems (Buehler, Lange, & Franck, 2007; Camisasca & Miragoli, 2014; Fosco & Grych, 2008; Lindsey, Colwell, Frabutt, & MacKinnon-Lewis, 2006). Another important property of the inter-parental discord is the content of the conflict. According to Grych and Fincham (Grych, 1998; Grych & Fincham, 1990, 1993), marital discord explicitly about child-rearing can be especially threatening or distressing for the children, by eliciting feelings of shame, blame, guilt and a greater motivation to intervene in marital conflict.

In literature, various mechanisms have been proposed to explain the link between inter-parental discord and child adjustment. More specifically, the Cognitive-Contextual Framework (Fosco, DeBoard, & Grych, 2007; Fosco & Grych, 2008; Grych & Fincham, 1990) emphasizes threat appraisals, self-blaming attributions and triangulation, as unique mechanisms that increase risk of maladjustment (Fosco & Grych, 2008). Threat appraisals reflect children’s evaluations of the personal relevance of inter-parental conflicts and the potential for harm to themselves or the family (Fosco et al., 2007; Fosco & Bray, 2016), that are strictly associated with children’s maladjustment (Camisasca, Miragoli, & Di Blasio, 2013, 2016b; Fosco & Feinberg, 2015; Fosco & Grych, 2008; Grych, Harold, & Miles, 2003). Self-blame appraisals may occur when children believe that the disagreement is caused by their behavior or if they feel responsible for ending or resolving the conflict (Fosco & Grych, 2008) with consequent associations to their maladjustment (Davies, Forman, Rasi, & Stevens, 2002; Fosco & Grych, 2008; Grych et al., 2003). Triangulation involves boundary violations, such that children become entangled in the inter-parental conflict and might feel caught in the middle (Bowen, 1978; Minuchin, 1974). From one side, parents could bring children into their disputes; from the other side, children may also feel caught in the middle or pressured to take sides, even if they do not become involved in the interactions (Amato & Afifi, 2006; Fosco & Grych, 2008; Franck & Buehler, 2007). Empirical evidence found that triangulation is a mechanism linking inter-parental conflict with children’s maladjustment (Buchanan & Waizenhofer, 2001; Fosco & Grych, 2008; Franck & Buehler, 2007; Gerard, Buehler, Franck, & Anderson, 2005).

Another conceptual model, the Emotional Security Hypothesis (Cummings & Davies, 2002; Davies & Cummings, 1994) underscores that the exposure to parental conflicts increases children’s insecurity about the marital relationship, manifested through children’s emotional distress, behavioral regulation of exposure to the conflict, and destructive internal representations of the family system (Davies et al., 2016). Literature showed that emotional distress was the most relevant dimension that uniquely mediated associations between inter-parental conflict and both internalizing and externalizing problems (Buehler et al., 2007; Davies, Cicchetti, & Martin, 2012; Davies & Cummings, 1998).

The studies above mentioned have substantially neglected to explore the possible associations among the content of the inter-parental discord and the children’s cognitive, emotional, behavioral processes and their adjustment, except for the studies of Shelton, Harold, Goeke-Morey, and Cummings (2006) and Koss et al. (2011). Shelton et al. (2006) examined how children’s coping efforts could vary as a function of the content of conflict (child or adult-related). Results showed that child-related conflict tactics characterized by physical aggression and verbal anger elicited children’s more severe mediation strategies, than adult-related conflict tactics. Similarly, Koss et al. (2011) showed that children reported more feelings of anger and sadness in response to the child-rearing disagreements, than other forms of conflicts. Moreover, their data illustrated that more intense scared feelings were associated with the escalating and unresolved conflicts, in comparison to the child-related conflict.

The present cross-sectional study, based on the suggestions of both the Cognitive Contextual Model (Grych & Fincham, 1990) and the Emotional Security Hypothesis (Davies & Cummings, 1994), was performed in order to advance knowledge of the specific role of adult-related versus child-related inter-parental conflict. More precisely, the study aimed to explore: 1) The distinct effects of adult-related *versus* child-related conflicts on children’s internalizing and externalizing behaviors; 2) the distinct effects of adult-related *versus* child-related conflicts on children’s cognitive appraisals (perceived threat and self blame), emotional distress, and triangulation; 3) the potential mediating role of threat, self-blame and triangulation, in the relationships between adult-related *versus* child-related conflicts and children’s internalizing and externalizing problems.

According to literature, we hypothesized that: (H1) both adult and child-related conflicts could be predictive of children’s internalizing and externalizing behaviors (Fosco & Grych, 2008; McCoy et al., 2009, 2013); (H2) child-related inter-parental conflict could be strongly associated with self-blame and triangulation (Grych & Fncham, 1993), rather than adult-related destructive conflict that could be more distressing for children (Davies et al., 2016; Koss et al., 2011). We also supposed (H3) that both forms of inter-parental conflict (adult and child-related conflict) could have indirect effects on children’s internalizing and externalizing behaviors, through perceived threat, self-blame, triangulation and emotional distress (Fosco & Grych, 2008).

## Method

### Participants

Participants were 75 Italian parents (mothers and fathers) and their 75 children (58% girls) ages 7–11 years (*M* = 9.35, *SD* = 1.33), recruited by four primary public schools located in Milan and in the Province of Milan. The children were noted as being the only child (36%), the first-born (32%), the second-born (27%), and the third-born (5%). The couples had been married 14 years on average (*SD* = 3.4). The mothers averaged 41.6 years of age (*SD* = 4.3), and the fathers averaged 43.9 years of age (*SD* = 5.2). Socio-Economic Status (SES) of participants’ families was assessed by asking for parents’ qualifications and jobs: 33% of participants were from middle-lower class, 48% from middle class, and 19% from middle-upper class.

### Procedure

We selected four primary public schools located in Milan and in the Province of Milan, mainly attended by families with an average socio-economic level. The schools were recruited by a standard procedure that included introductory meetings with school principals and letters to the parents, describing the goals and procedures of the study. One hundred and twelve families (convenience sampling) were approached, of whom spontaneous and unpaid consent to participate was obtained from 88 parents (73.3%). Parents signed consent forms, for themselves and their children, that described the project and its goals, the voluntary nature of participation, and the confidentiality of the data collected. Each participant could also have the possibility to give up the research at any time without any explanation and could request a personal meeting with the referent researcher before and at the end of the completion of the questionnaires.

The school-aged children were approached in school classrooms during regular class time at the convenience of participating teachers. Two research assistants, trained by the authors, during their research internship of 6 months, conducted the administration procedure. The measures were administered with a random order and research assistants gave assistance to children if they had difficulty in understanding any of the questions (i.e., reading the items and word definitions). Moreover, in case of children’s distress, research assistants could stop the procedure and relieve the children. Completion of the measures examined in the present study took approximately 20 minutes. Packets consisting of self-report measures (see the Measures Section) were delivered to parents. Measures were accompanied by a letter describing the modalities for their self-administering, in which mothers and fathers were asked to fill in the forms independently, without sharing their answers, and to return all the questionnaires by the next 2 months to the schools. For thirteen families (nine fathers and four mothers) we found missing and incomplete data and we deleted these families from the data set. We therefore obtained complete data for 75 parents and their children. Ethical approval was obtained from the Ethics Committee of the Catholic University of Milan for the project Inter-parental conflict and child adjustment, in April 2013.

### Measures

#### Adult-Related Conflict

Children completed the *Conflict Properties Scale* (19 items) from the *Children’s Perceptions of Inter-Parental Conflict Scale* (CPIC; Grych, Seid, & Fincham, 1992; the back translation was approved by the Author), which reflects conflict that occurs regularly, involves higher levels of hostility, and is poorly resolved and is composed of three sub-scales: Frequency, Intensity, and Resolution. Children rated each item, using a 3-point scale (“true,” “sort of true,” “false”). Sample items include: “I often see my parents arguing” (Frequency), “My parents get really mad when they argue” (Intensity), and “Even after my parents stop arguing, they stay mad at each other” (Resolution). This scale has demonstrated excellent reliability and validity in past research (Fosco & Grych, 2008) and in the current sample (α = .83). It has also been used with Italian-speaking populations with a good internal reliability (α = .82; Camisasca et al., 2016b).

Both parents completed the *Dyadic Satisfaction Scale* (10 items) of the *Dyadic Adjustment Scale* (DAS; Spanier, 1976; Italian validation by Gentili, Contreras, Cassaniti, & D’Arista, 2002). In order to gain their perspective about the frequency of quarrels, disagreements, conflicts, three items of the Dyadic Satisfaction Scale were considered, and specifically: Item 17 (How often do you or your mate leave the house after a fight?); item 21 (How often do you and your partner quarrel?); item 22 (How often do you and your mate “get on each other’s nerves”). Both parents rated the three items with a 6-point response format ranging from 5 (All the Time) to 0 (Never). This measure has demonstrated excellent reliability and validity in past research (α = .90; Camisasca, Miragoli, Caravita, & Di Blasio, 2015; Camisasca, Miragoli, Milani, & Di Blasio, 2016) and in the current sample (α = .80). Following the suggestions of Fosco and Grych (2008), children’s, mothers’, and fathers’ reports were converted into *z*-scores and then the mean score was computed to create a single score of Adult-Related Inter-parental Conflict.

#### Child-Related Conflict

Inter-parental conflict was assessed via self-reports by children and both of their parents. Children completed the *Conflict Child-Related Content,* from the Children’s Perceptions of Inter-parental Conflict scale (CPIC; Grych et al., 1992), which assesses the content of the conflict that specifically pertains to children’s rearing or behaviors. Children rated the 4 items as either “true”, “sort of true” or “false”. Sample items include: “My parents’ arguments are usually about me”, “My parents usually argue or disagree because of things that I do”. This scale has demonstrated good reliability and validity in past research (Grych et al., 1992) and yielded excellent reliability in the current sample (α = .86).

Both parents also completed the *Children’s Exposure to Conflict Scale* of the Coparenting Relationship Scale (CRS; Feinberg, Brown, & Kan, 2012; the back translation was approved by the Author), to gain their perspective about children’s exposure to inter-parental conflict, concerning the rearing of the child (5 items). Sample items include: “How often in a typical week, when all 3 of you are together, do you argue with your partner about your child, in the child’s presence?”

This scale has demonstrated excellent reliability and validity in past research (α = .82; Camisasca et al., 2016b; Camisasca, Miragoli, Di Blasio & Feinberg, 2018) and in the current sample (α = .80). Following the suggestions of Fosco and Grych (2008), children’s, mothers’, and fathers’ reports were converted into *z*-scores and then computed the mean score to create a single score of Child-Related Inter-parental Conflict.

#### Appraisals of Inter-Parental Conflict

The *Perceived Threat* and *Self-Blame* sub-scales from the CPIC (Grych et al., 1992) were used to assess children’s appraisals of inter-parental conflict. The six-items of the *Perceived Threat* sub-scale assess the level of threat reported by respondents when inter-parental conflict occurs. The nine-items of the Self-Blame scale measure the respondents’ tendency to blame themselves for these disagreements. Sample items include: “My parents blame me when they have arguments”; “Even if they don't say it, I know I'm to blame when my parents argue.” These scales have demonstrated adequate reliability and validity in past research (Fosco & Grych, 2008; Grych et al., 1992) and in the current sample (perceived threat: α = .78; self-blame: α = .77). They have also been used with Italian-speaking populations with a satisfactory internal reliability (α = .72; Camisasca et al., 2016b).

#### Triangulation

Children’s triangulation into parental conflicts was assessed using the *Triangulation* sub-scale of the CPIC (Grych et al., 1992). This eight-item sub-scale assesses a wide range of triangulation behaviors, capturing the extent to which children feel involved in, caught in the middle of, or drawn into cross-generational coalitions during inter-parental disagreements. Sample items include: “My mom wants me to be on her side when she and my dad argue”; “I feel like I have to take sides when my parents have a disagreement”. This scale has demonstrated adequate reliability and validity in past research (Fosco & Grych, 2008) and in the current sample (α = .76).

#### Children’s Distress Reactions to Inter-Parental Conflict

To assess children’s distress from the inter-parental conflict, children completed one sub-scale of the Security in the Inter-Parental Subsystem (SIS; Davies et al., 2002). The *Distress sub-scale* includes 12 items with a 4-point response format and includes examples of multiple, prolonged, and dysregulated expressions of emotion and distress. Sample items include: “When my parents argue, I feel scared”, “I hit, kick, slap, or throw things at people in my family”. This scale yielded adequate reliability in the current sample (α = .79) and it has also been used with Italian-speaking populations with a satisfactory internal reliability (α = .77; Camisasca, Miragoli, & Di Blasio, 2016a).

#### Internalizing and Externalizing Behaviors

Both parents completed the *Child Behavior CheckList* (CBCL/4-18; Achenbach, 1991; Italian version by Frigerio, 2001) that is one of the most extensively used measures of children’s internalizing and externalizing problems. Parents rated the 113 items of the measure as 0 (not true), 1 (somewhat or sometimes true), or 2 (very true or often true). The *Internalizing scales* reflect a pattern of maladjustment characterized by social withdrawal or shyness and symptoms of depression or anxiety. The *Externalizing scales* capture children’s maladjustment characterized by aggression and defiance. In our study excellent reliability was found for internalizing (for mothers: α = .88 and for fathers: α = .84) and externalizing problems (for mothers: α = .86 and for fathers: α = .88). Following the suggestions of Fosco and Grych (2008), mothers’ and fathers’ reports were converted into *z*-scores and then the mean score was computed to create a single *Parent Index* of internalizing and externalizing problems.

### Data Analysis Strategy

Descriptive statistics were computed for all the variables. *t*-test analyses were computed in order to verify the presence of any differences of children’s age groups (7–8 and 9–11 years) in the distribution of the variables investigated. Pearson’s r correlations were used to investigate the associations between the variables. The proposed mediation model was examined using the Process Macro for SPSS (Hayes, 2013), applying Model 4 with 5000 bias-corrected bootstrap samples. The bias-corrected bootstrapping resampling method is particularly suitable for small samples (Hayes, 2012). Our preference is based on the fact that other methods for testing indirect effects assume a standard normal distribution when calculating the p-value for the indirect effect, whereas bootstrapping does not assume normality of the sampling distribution (Hayes, 2012). In addition, the bootstrap method repeatedly samples from the dataset, estimating the indirect effect with each resampled dataset. This process is repeated thousands of times, producing bias-corrected accelerated confidence intervals for the indirect effect.

## Results

### Descriptive and Correlational Analyses

Means and standard deviations of all variables used in the present study are presented in Table 1. Regarding the variables investigated, the means scores, for mothers, fathers and children, were similar to those obtained in other Italian and international studies with normative samples (Camisasca et al., 2016; Feinberg et al., 2012; Frigerio, 2001) and were placed within normal limits.

**Table 1:** Descriptive Statistics, Mean, Standard Deviation, and Min-Max Scores

Variable	*M*	*SD*	Min-Max
Adult-related conflict (Mothers: DAS)	11.12	1.84	3–14
Adult-related conflict (Fathers: DAS)	11.16	1.83	6–15
Adult-related conflict (Children: CPIC)	10.48	6.90	0–35
Child-related conflict (Mothers)	0.91	0.75	0–3
Child-related conflict (Fathers)	0.88	0.80	0–3
Child-related conflict (Children: CPIC-Content)	2.04	1.60	0–11
Perceived threat (CPIC)	3.78	3.11	0–11
Self-blame (CPIC)	2.40	2.11	0–10
Triangulation (CPIC)	4.23	3.16	0–11
Emotional distress (SIS)	17.54	5.56	9–34
Internalizing behaviors (CBCL)	6.55	4.51	0–23
Externalizing behaviors (CBCL)	6.88	4.61	0–24

The *t*-test analyses showed that there were not significant differences between the two age groups (7–9 and 10–11 years) in the distribution of the variables explored, *t*(68) from 0.18 to –1.48; *p* > .05).

The correlation analyses showed that the variables were correlated (see Table 2). In particular, both adult- and child-related conflicts were correlated with internalizing and externalizing behaviors (*r* from .23 to .52) and to cognitive appraisals, distress reactions and triangulation (*r* from .23 to .59). Cognitive appraisals, distress reactions and triangulation were also significantly associated with internalizing and externalizing behaviors (*r* from .28 to .71).

**Table 2:** Correlational Analyses

Variable	1	2	3	4	5	6	7	8
1. Adult-related conflict	–							
2. Child-related conflict	.32**	–						
3. Perceived threat	.38**	.43**	–					
4. Self-blame	.23*	.59**	.42**	–				
5. Emotional distress	.43**	.35**	.62**	.29*	–			
6. Triangulation	.32**	.41**	.54**	.39**	.29*	–		
7. Internalizing behaviors	.40**	.45**	.70**	.43**	.48**	.51**	–	
8. Externalizing behaviors	.23*	.51**	.36**	.50**	.33**	.28*	.56**	–

*p < .05. **p < .01.

### Test of Mediation

We performed mediational analyses using the Process Macro for SPSS (Hayes, 2013), applying Model 4 with 5000 bias-corrected bootstrap samples. In the mediational model, adult- *versus* child-related conflicts were added as predictors, internalizing and externalizing behaviors as outcomes, and the perceived threat, self-blame, distress reactions and triangulation as mediators (see Table 3).

**Table 3:** Indirect Effects of Children’s Cognitive, Emotional and Behavioral Processes on Internalizing and Externalizing Symptoms

Predictor	β	*SE*	Bootstrap 95% CI		Model	
			*LL*	*UL*	*R*^2^	*p*
DV: Internalizing behaviors						
Total effects					.28	< .001
Adult-related conflict	.13*	.04				
Child-related conflict	.41**	.12				
Direct effects					.01	> .05
Indirect effect via mediator *Perceived threat*					
Adult-related conflict	.06*	.03	.00	.14		
Child-related conflict	.20*	.08	.08	.41		
Indirect effect via mediator *Self-blame*					
Adult-related conflict	–.01	.00	–.02	.00		
Child-related conflict	.07	.07	–.07	.22		
Indirect effect via mediator *Emotional distress*						
Adult-related conflict	.00	.02	–.02	.05		
Child-related conflict	.01	.03	–.04	.11		
Indirect effect via mediator *Triangulation*						
Adult-related conflict	.01	.01	–.00	.05		
Child-related conflict	.05	.04	–.01	.17		
DV: Externalizing behaviors						
Total effects					.27	< .001
Adult-related conflict	.02	.05				
Child-related conflict	.62*	.13				
Direct effects					.05	> .05
Indirect effect via mediator *Perceived threat*						
Adult-related conflict	.00	.02	–.03	.07		
Child-related conflict	.01	.07	–.11	.19		
Indirect effect via mediator *Self-blame*						
Adult-related conflict	–.00	.01	–.05	.02		
Child-related conflict	.21	.09	.05	.41		
Indirect effect via mediator *Emotional distress*						
Adult-related conflict	.02	.02	–.01	.08		
Child-related conflict	.03	.03	–.02	.13		
Indirect effect via mediator *Triangulation*						
Adult-related conflict	.00	.01	–.03	.02		
Child-related conflict	–.00	.02	–.05	.06		

*Note.* CI = confidence interval; LL = lower limit; UL = upper limit.

*p < .05. **p < .01.

Regarding internalizing behaviors, only both the total effects of adult-related and child-related conflict were significant (adult-related: β = .13, *p* < .05; child-related: β = .41, *p* < .01; *c path*). The effects of the adult-related conflicts were significant on children’s perceived threat (β = .12, *p* < .05; *a path*) and distress reactions (β = .27, *p* < .01; *a path*), while the effects of the child-related conflicts were significant on all the mediators: children’s perceived threat (β = .39, *p* < .01; *a path*), self blame (β = .45, *p* < .01; *a path*), distress (β = .46, *p* < .01; *a path*), and triangulation (β = .39, *p* < .01; *a path*). When the effects of the mediators (perceived threat, self blame, distress, and triangulation) were controlled, the direct effects of adult-related and child-related conflict on children’s internalizing problems were not significant (adult-related: β = .05, *p* > .05; child-related: β = .08, *p* > .05; *c’ path*). Moreover, children’s perceived threat mediated the relationships between both adult and child-related conflict and children’s internalizing problems (adult-related: β = .06, 95% CI [.00, .14]; child-related: β = .20, 95% CI [.08, .41]).

Regarding externalizing behaviors, only the total effect of the child-related conflict was significant (β = .62, *p* < .01; *c path*). As above, the effects of the adult-related conflicts were significant on children’s perceived threat (β = .12, *p* < .05; *a path*) and distress reactions (β = .27, *p* < .01; *a path*), while the effects of the child-related conflicts were significant on all the mediators: children’s perceived threat (β = .39, *p* < .01; *a path*), self blame (β = .45, *p* < .01; *a path*), distress (β = .46, *p* < .01; *a path*), and triangulation (β = .39, *p* < .01; *a path*). When the effects of the mediators (perceived threat, self blame, distress, and triangulation) were controlled, the direct effects of adult and child-related conflicts on children’s externalizing problems were not significant (adult-related: β = .00, *p* > .05; *c’ path*; child-related: β = .29, *p* > .05; *c’ path*). Moreover, children’s self blame mediated the relationship between child-related conflict and children’s externalizing problems (β = .21, 95% CI [.05, .41]).

## Discussion

Although many studies have explored associations between marital conflict and child adjustment (Fosco & Grych, 2008; McCoy et al., 2013), the interest of how the distinct forms of inter-parental conflict (adult-related *versus* child-related) may differentially impact children’s cognitive, emotional and behavioral processes and their psychological adjustment is a new direction of research. Our results, in line with expectations found correlations among the two predictors (adult-related *versus* child-related conflict), the mediators (children’s cognitive, emotional, and behavioral processes), and the outcomes (children’s internalizing and externalizing behaviors). More precisely, higher levels of both adult-related and child-related conflicts correlated with higher levels of children’s perceived threat and self blame, distress reactions and triangulation, and with more children’s internalizing and externalizing behaviors. Furthermore, the mediational analyses indicated distinct predictive effects of adult-related *versus* child-related conflicts on children’s adjustment and their cognitive, emotive, and behavioral processes. More precisely, contrary to our hypothesis (H1), adult-related conflict was strictly related to only children’s internalizing behaviors, while child-related conflict to both children’s internalizing and externalizing behaviors. Moreover, as hypothesized (H2), adult-related conflict predicted perceived threat and emotional distress while child-related conflict predicted children’s self-blame and triangulation. Moreover, unlike expectations, child-related conflict also predicted children’s perceived threat and emotional distress.

Consistent with literature (Camisasca et al., 2017; Goeke-Morey et al., 2003; Koss et al., 2011), our results reported that adult-related conflict, connoted by chronic, unresolved and intense levels of hostility, undermined children’s sense of security, leaving them threatened and worried and emotionally distressed. Moreover, adult-related conflicts, connoted by chronic, intense and unresolved disagreements, predicted children’s internalizing problems, through the mediation of the perceived threat. These data are consistent with longitudinal studies that outlined how children’s perceived threat was a significant risk factor for the insurgence of internalizing problems (Fosco & Feinberg, 2015; Grych et al., 2003). We could explain our data, by suggesting that children’s exposed to higher levels of adult-related inter-parental conflicts could be more vulnerable to internalizing behaviors through an amplification of their worries and scared feelings, that conflict will escalate, threatening the family’s existence (Fosco & Bray, 2016; Fosco et al., 2007).

Regarding child-related conflicts, the results were in line with previous literature (Koss et al., 2011; Schelton et al., 2006), which showed how children exposed to marital disagreements, explicitly about child-rearing, could develop feelings of shame, blame and guilt. In turn, these feelings could promote children’s self-blame appraisals connoted by the idea of being responsible for ending or resolving the conflict and a greater vulnerability to triangulation. Moreover, our results indicated that child-related conflict could also scare and threaten the children, by increasing their levels of perceived threat and distress reactions. In other words, children could believe that child-related conflict could also cause damage to self or others, or to the family’s survival, with consequent emotional insecurity and distress reactions. These data supported the suggestions of the literature (Grych, 1998, Grych & Fincham, 1990, 1993; Koss et al., 2011) that marital disagreements explicitly about children or child-rearing can be especially threatening or distressing for children, by eliciting feelings of sadness, anger, shame, blame, guilt and a greater motivation to intervene in marital conflict.

Data also illustrated that child-related conflict, through the mediation of perceived threat and self-blame appraisals, predicted both children’s internalizing and externalizing behaviors, while adult-related conflict, through the mediation of perceived threat, predicted only children’s internalizing behaviors. In other words, contrary to our expectations (H3), our data seemed to strengthen the negative effects of child-related conflict, compared to adult-related conflict, on children’s adjustment. The results that child-related conflict predicted children’s internalizing problems, through the mediation of perceived threat are consistent with the literature that outlined how this type of conflict could promote feelings of sadness, worries and guilt (Grych & Fincham, 1993; Koss et al., 2011) and with the empirical evidence that indicated robust relations between perceived threat and internalizing problems (Camisasca et al., 2017; Fosco & Feinberg, 2015; Grych et al., 2003). Moreover, our results seemed to strengthen those of previous studies that outlined how child-related conflict could promote anger reactions in children (Koss et al., 2011) and how self-blame is uniquely associated with externalizing (but not internalizing) behaviors (Davies et al., 2002; Fosco & Grych, 2008; Shelton & Harold, 2008).

In order to explain these results, we can suppose that feeling responsible for inter-parental conflict provided children with a sense of coping efficacy and perceived control over conflict, which increases the likelihood of involvement (Grych, 1998; Schelton & Harold, 2008). When children involve themselves in conflict, they may use coercive and aggressive tactics in an attempt to distract or bring an end to parents’ marital arguments, with consequent externalizing problems (Schelton & Harold, 2008). Moreover, we can also suggest that some children, who feel blame and responsibility for parents’ arguments, could be unable (or perhaps prevented from) intervening and may express their distress and frustration in the form of anger and acting out. Previous literature suggests that painful feelings of shame could elicit defensiveness, anger, and overt aggression (Stuewig, Tangney, Heigel, Harty, & McCloskey, 2010). On the basis of these suggestions, we can therefore suppose that children’s self-blame appraisals, due to child-related conflicts, could also transform their painful feelings into maladaptive responses, such as hostility, anger and sometimes aggression (externalizing behaviors).

Taken together, our results showed that: 1) high levels of adult-related inter-parental conflict promoted children’s internalizing behaviors, through the mediation of perceived threat appraisals; 2) high levels of child-related inter-parental discord promoted both children’s internalizing and externalizing behaviors, through the mediation of perceived threat and self-blame appraisals. In conclusion, child-related conflict, compared to adult-related conflict, assumed the role of a particularly significant risk factor for the children’s adjustment, making children vulnerable to the onset of both internalizing and externalizing problems.

Several limitations of this study should be noted. First, the real direction of relations among variables examined in this study cannot be empirically evaluated, because the research design of this study is cross-sectional. The nature of our cross-sectional data cannot explicitly identify directional effects or causal links, while longitudinal data could provide stronger evidence of directionality or causality. The small number of participants may limit the generalizability of our results, even though this limitation was partly overcome by the use of the bootstrapping procedure for the mediational analyses. Another limitation of the study is the use of only self-report data. It is now known self-report measures assessing sensitive information can be subject to social desirability and can inflate some of the associations among variables due to shared method variance. We could also outline that the responses of the first and second graders children may bias the study results depending on how research assistants helped these participants. Indeed, Grych, Seid, and Fincham (1992) stated that: “The language on the CPIC makes it appropriate for use with children from third grade through junior high school, but children's perceptions and interpretations of marital conflict are likely to change with age, and consequently the reliability, validity, and factor structure of the CPIC may differ across developmental levels” (p. 570). Moreover, another limitation of the study is that measures administered included many more items than actual participants in the study. Future research should use a multi-method approach, including observational methods, teacher-reports, and interviews in which parents can describe their marital relationships. In addition, our sample was composed of Italian parents who were predominantly well-educated and of middle-class. Not significant associations were showed between parents’ age, gender and SES and the variables investigated (r from .00 to .18). Replications of our findings with a more heterogeneous samples would foster generalization of findings to a broader population.

Despite these limitations, our findings helped to advance our understanding of the relationships between distinct forms of marital conflict and children’s adjustment, by examining the cognitive, emotional, and behavioral underlying processes.
